# Cancer biotherapy: review and prospect

**DOI:** 10.1007/s10238-024-01376-2

**Published:** 2024-05-27

**Authors:** Qi Liu, Hu Ma

**Affiliations:** 1 Zunyi Medical University, Zunyi, Guizhou, 563000 China; 2 Department of Thoracic Oncology, The Second Affiliated Hospital of Zunyi Medical University, Guizhou, 56300 Zunyi China

**Keywords:** Tumors, Biological therapy, Immunotherapy, Gene therapy

## Abstract

Malignant tumors pose a grave threat to the quality of human life. The prevalence of malignant tumors in China is steadily rising. Presently, clinical interventions encompass surgery, radiotherapy, and pharmaceutical therapy in isolation or combination. Nonetheless, these modalities fail to completely eradicate malignant tumor cells, frequently leading to metastasis and recurrence. Conversely, tumor biotherapy has emerged as an encouraging fourth approach in preventing and managing malignant tumors owing to its safety, efficacy, and minimal adverse effects. Currently, a range of tumor biotherapy techniques are employed, including gene therapy, tumor vaccines, monoclonal antibody therapy, cancer stem cell therapy, cytokine therapy, and adoptive cellular immunotherapy. This study aims to comprehensively review the latest developments in biological treatments for malignant tumors.

## Introduction

**Fig. 1:**
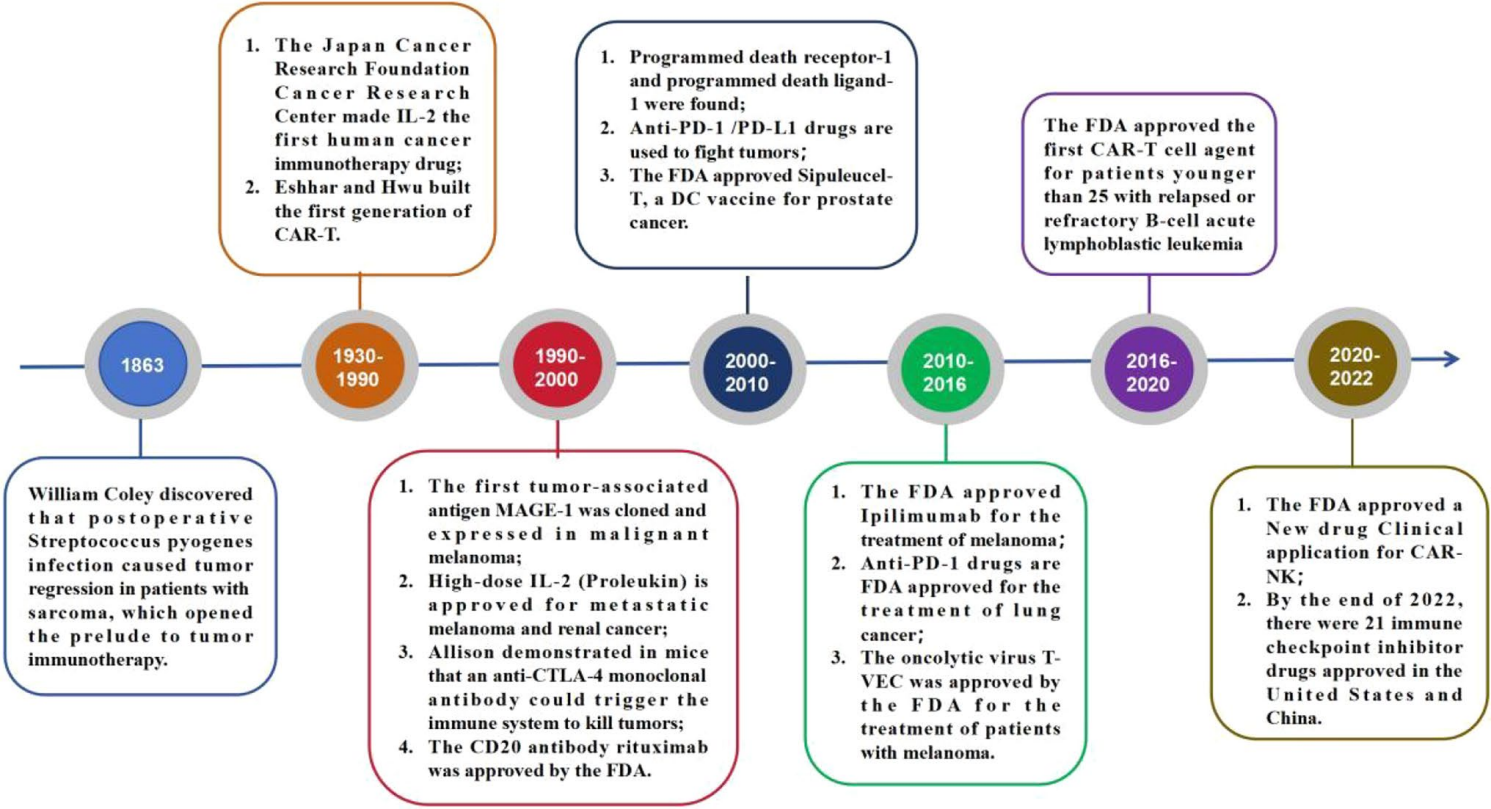
A hundred years of development of cancer biotherapy

**Fig. 2:**
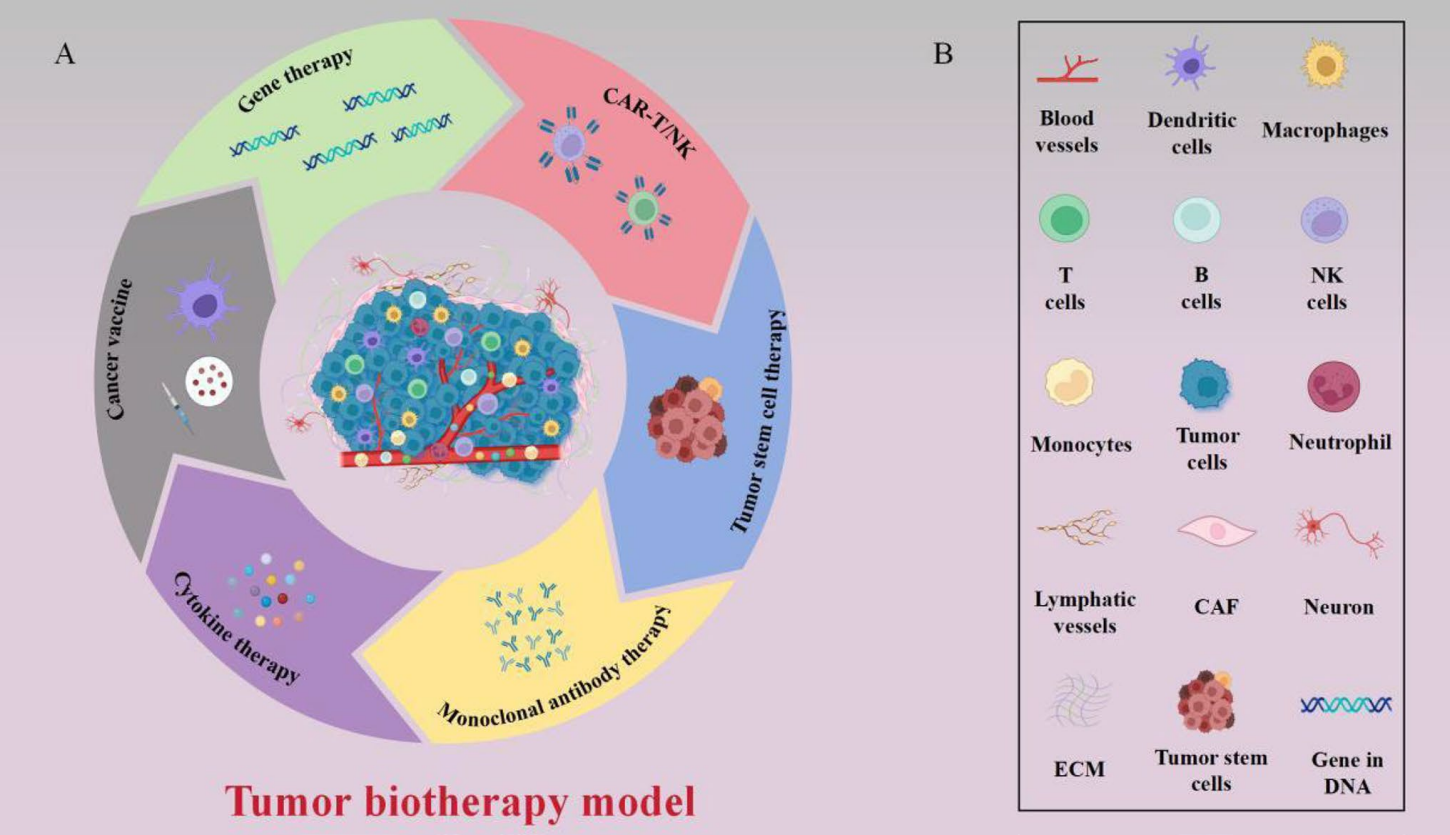
Main treatment mode of tumor biology. **A** Biotherapy for malignant tumors encompasses diverse methodologies, including genes therapy, tumor vaccines, monoclonal antibodies (mAbs) therapy, stem cells (SC) therapy, cytokines therapy, and adoptive cell therapies (ACT); B) There are blood vessels, immune cells, fibroblasts, bone marrow-derived inflammatory cells, various signaling molecules and extracellular matrix in the tumor microenvironment

As a result of rapid socio-economic development and environmental changes, malignant tumors have emerged as a significant health concern, posing a grave threat to human life. The overall prevalence of malignant tumors is on the rise in our country. Recent statistics predict an estimated 1.95 million new cases and 0.60 million deaths from malignant tumors in the United States by 2023 [1]. Notably, lung cancer (LUNG), colorectal cancer (COAD), gastric cancer (GC), hepatocellular carcinoma (HCC), and breast cancer (BRCA) are the most common and contribute prominently to the escalating mortality rates. Malignant tumors possess an inherent characteristic of unrestricted proliferation, making their complete eradication through surgery, radiotherapy (RT), or chemotherapy alone highly challenging. Interestingly, tumor biotherapy had already entered clinical practice a century ago (Fig.1) [2–13]. However, limited advancements in biotechnology hindered its widespread clinical application back then. Subsequently, with extensive research on the molecular mechanisms underlying tumor occurrence and development and the rapid progress in biotechnology, tumor biotherapy has emerged as a novel and fourth primary treatment modality, complementing surgery, RT, and chemotherapy [14]. Tumor biotherapy utilizes biological engineering techniques and agents to cultivate and amplify immune cells harvested from patients. Subsequently, these bolstered cells are reintroduced into patients' bodies to stimulate and enhance their own immune system, ultimately fostering the fight against malignant tumors [15]. Presently, biotherapy for malignant tumors encompasses diverse methodologies, including genes therapy, tumor vaccines, monoclonal antibodies (mAbs) therapy, stem cells (SC) therapy, cytokines therapy, and adoptive cell therapies (ACT) (Fig.2). This article aims to provide a comprehensive overview of the recent research advancements in biotherapy for specific malignant tumors.

## Genes therapy

**Fig. 3:**
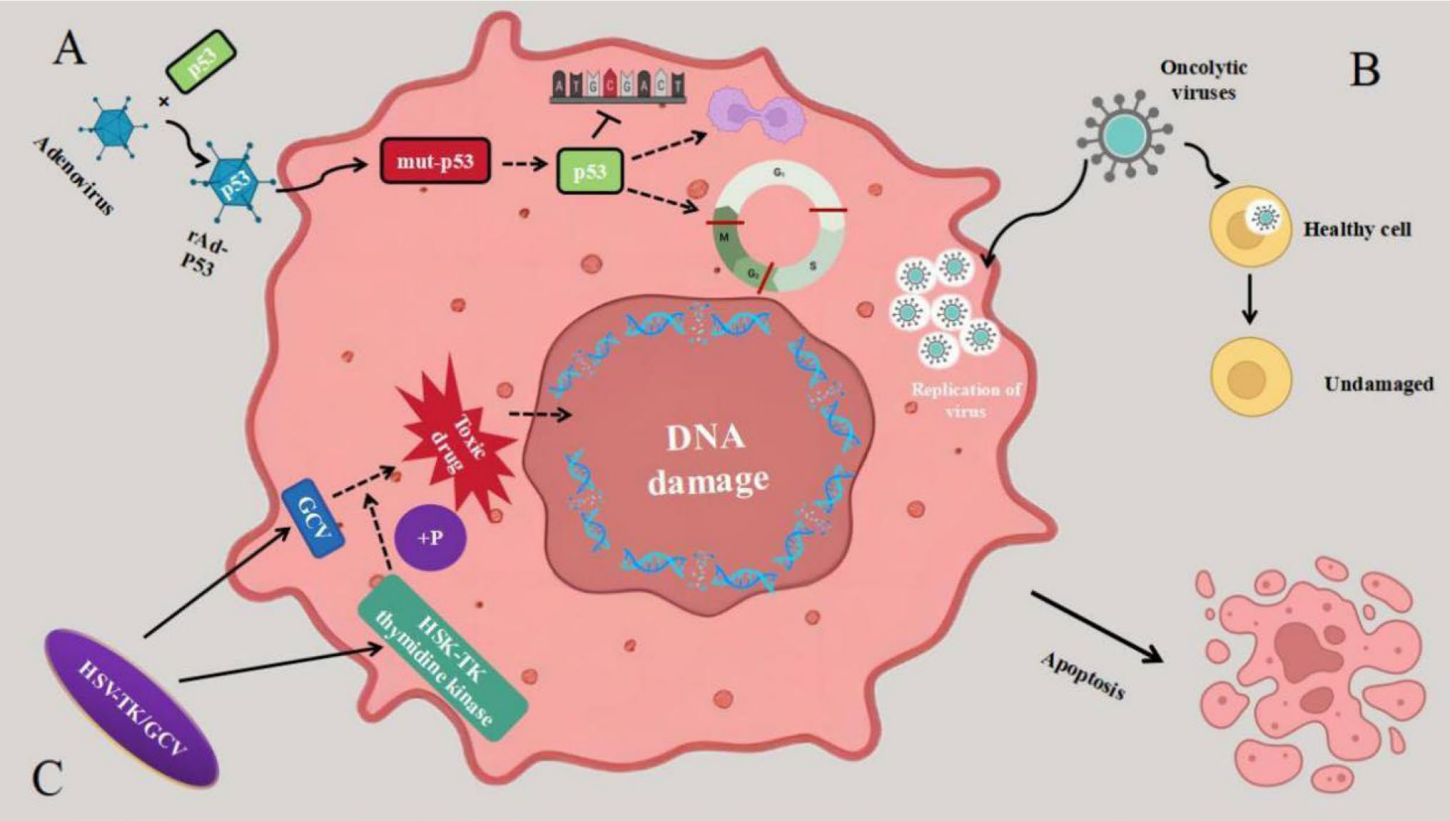
Mechanisms of gene therapy in cancer. A Tumor suppressor genes therapy aims to introduce normal oncogenes into tumor cells using vectors. This procedure replaces the mutated oncogenes with functional ones, restoring average growth and development in the tumor cells. **B** Suicide genes therapy, or enzyme-mediated prodrug therapy, is a dynamic process involving two steps. Initially, the genetic material of tumor cells is altered by introducing non-harmful enzyme genes (referred to as suicide genes) that code for enzymes responsible for activating prodrugs. Subsequently, these enzymes facilitate the conversion of benign drugs into toxic substances, eradicating the tumor cells. **C** Oncolytic viruses are a replicating viruses that specifically target and replicate within tumor cells in the tumor microenvironment (TME), leaving normal cells unharmed

Gene therapy is a biomedical technology that involves the insertion of normal or therapeutic exogenous genes into target cells. The aim is to repair or replace defective genes in these cells, ultimately treating diseases. Current tumor gene therapy includes tumor suppressor gene therapy, suicide gene therapy and oncolytic virus gene therapy (Fig.3).

### Tumor suppressor genes therapy

Tumor suppressor genes (TSGs) can be found in normal cells and play a crucial role in inhibiting cell growth and preventing the development of cancer. The discovery of these genes dates back to the 1960s when H. Harris investigated cells with heterozygosity and their oncogenic properties. TSGs have been found to exert their anti-tumor effects through their influence on genome integrity, cell cycle regulation, and cell proliferation control [16]. Currently, scientists have identified approximately twenty oncogenes, which include adenomatous polyposis coli (APC), ataxia telangiectasia mutated (ATM), AT-rich interaction domain 1A (ARID1A), p53, phosphatase, and tensin homolog deleted on chromosome ten (PTEN), and fragile histidine triad gene (FHIT) [17]. Oncogene therapy, a therapeutic approach, aims to introduce normal oncogenes into tumor cells using vectors [18]. This procedure replaces the mutated oncogenes with functional ones, restoring average growth and development in the tumor cells. Researchers commonly employ viral vectors to re-express the normal p53 oncogene, particularly an adenoviral vector called recombinant adenovirus human *p53 (*rAd-*p53*). This vector facilitates the introduction of the recombinant tumor suppressor p53 (TP53) gene into specific cancer cells. An exemplary case of this approach is Gendicine. This gene therapy product gained approval from the National Medical Products Administration (NMPA) in 2003 for treating head and neck squamous cell carcinoma (HNSC) [19]. In a study by Xiao et al., they combined rAd-*p53* gene therapy with RT and hyperthermia to treat advanced soft tissue sarcoma (STS). The researchers observed that the group treated with p53 had a disease control rate (DCR) of 83.33%, with only a 16.67% disease progression rate [20]. Subsequent investigations have also shown promising outcomes for rAd-*p53* in treating LUNG, BRCA, thyroid cancer (THCA), and other tumors [21–25]. It can be used as a standalone therapy or in combination with other treatments. Nevertheless, the intricate internal environment of the tumor and the limited advancement of TSGs transfer technology restrict the therapeutic gene's impact solely on the cancer cells that receive it, leaving other cancer cells lacking the tumor suppressor genes to continue growing [26]. The difficulty in fully penetrating the entire tumor tissue poses a significant risk of tumor recurrence. As a result, the efficacy and safety of tumor suppressor genes therapy using gene therapy methods must be thoroughly studied and evaluated. Further investigation is necessary to determine the effectiveness and safety of this approach.

### Suicide genes therapy

Suicide genes therapy, or enzyme-mediated prodrug therapy, is a dynamic process involving two steps. Initially, the genetic material of tumor cells is altered by introducing non-harmful enzyme genes (referred to as suicide genes) that code for enzymes responsible for activating prodrugs. Subsequently, these enzymes facilitate the conversion of benign drugs into toxic substances, eradicating the tumor cells [27]. Well-known combinations of suicide genes and prodrugs, such as herpes simplex virus thymidine kinase (HSV-TK)/ganciclovir (GCV) and cytosine deaminase (CD)/5-fluorocytosine (5-FC), have been extensively studied [28, 29]. The bystander effect is a crucial determinant of the success of suicide gene therapy. This phenomenon extends the drug's cytotoxic impact beyond the genetically modified cells, forming a lethal zone around them [30]. However, suicide gene therapy utilizing HSV-TK encounters various drawbacks, including suboptimal kinetics for prodrug activation, limited mechanisms of cell toxicity, the emergence of drug resistance, and the requirement of preemptive GCV administration in clinical settings. These factors hinder the widespread adoption of this therapeutic approach. To underscore the potential of suicide gene therapy, scientists conducted experiments involving colorectal xenograft tumors in mice [31]. Specifically, they injected an adenovirus vector carrying HSV-TK into one liver lobe, subsequently administering GCV. Intriguingly, complete regression of tumors was observed not only in the injected lobe but also in the non-injected lobe. Moreover, researchers noticed the infiltration of CD8^+^ T cells and initiation of an inflammatory response surrounding the tumor in the non-injected lobe, signifying an immune reaction. In other studies, involving glioblastoma (GBMLGG) patients, similar results have been observed with HSV-TK/GCV gene therapy. These patients exhibited elevated levels of interleukin-12 (IL-12) and interferon-γ (IFN-γ), indicating that the immune system was activated and anti-tumor antigens were released into the bloodstream after tumor cell death [32]. The CD/5-FC system has also demonstrated effectiveness against various cancers, including bladder cancer (BLCA), rectal cancer (READ), brain lower-grade glioma (LGG), and melanoma [33–36]. However, one major challenge in using suicide genes therapy for solid tumors is the limited ability of the therapeutic genes carrier to reach the tumor's core cells [37]. This can lead to a decrease in the expression of suicide genes, either in quantity or duration. The conversion rate from precursor to active drugs may significantly impact the treatment's effectiveness. For the clinical application of suicide genes therapy to be improved, it is vital to target the localization of genes in malignant or metastatic cancer cells while also achieving substantial bystander effects. However, minimizing any harm caused to non-cancer cells surrounding the tumor is equally essential.

### Oncolytic virus genes therapy

Oncolytic Virus (OV) is a specialized type of virus capable of targeting and destroying tumor cells selectively. Through its unique replication mechanism, it can also trigger the body's immune system to generate a response against the tumor [38]. This therapeutic approach offers a multimodal strategy for precisely and effectively eliminating malignant cells. The field of OVs has seen significant advancements in both diagnosis and treatment in domestic and international arenas. At present, four types of OVs treatment drugs have been approved and are available in the global market. These include RIGVIR (produced by Sia Latima in Latvia) for melanoma and other malignant tumors, Ankeri H101 (produced by Shanghai Sanwei) for advanced or recurrent HNSCs, T-VEC (developed by Biovex, a subsidiary of Amgen) for advanced melanoma, and Delytact (produced by Daiichi Sankyo in Japan) for GBMLGG and other cancers [39–42]. Recent studies have shown the efficacy of combining OVs with chemotherapy in treating solid tumors like non-small lung cancer (NSCLC) and BC [43, 44]. In addition, combining OVs with RT has shown promise in treating HNSC and LGG [45, 46]. Similarly, the combination of OVs with immunotherapy has yielded positive results in the treatment of malignant melanoma [47–49]. These findings suggest that integrating OVs with other treatment modalities can enhance therapeutic outcomes and potentially provide new avenues for cancer treatment. However, antiviral treatments face a significant issue: the limited efficacy of current delivery techniques. To address this, scientists have developed a multimodal strategy using OVs that capitalizes on the immune response against viruses and redirects it toward a tumor-associated antigen (TAA) that is common among tumors [50]. This strategy presents a powerful in situ vaccination method against tumors, targeting multiple tumor antigens in cases of low mutational burden to enhance the anti-tumor effects. Moreover, recent investigations have discovered that combining alum sulfate with vesicular stomatitis virus, an oncolytic virus, can notably elevate the levels of IFN-γ and interleukin-6 (IL-6), thereby enhancing the response of tumor antigen-specific T cells [51]. Nevertheless, the combination of OVs therapy and immune checkpoint inhibitors (ICI) has not exhibited significant clinical benefits. One of the primary hurdles is the accurate targeting of the virus to the tumor, which can be impeded by the presence of the preexisting immune system [52]. Another challenge involves optimizing the concurrent use of OVs with chemotherapy or immunotherapy drugs to achieve more favorable and consistent results. However, if these obstacles can be effectively overcome, OVs in tumor biotherapy possess the potential to emerge as an optimal and painless treatment alternative for cancer patients.

## Tumor vaccines

**Fig. 4:**
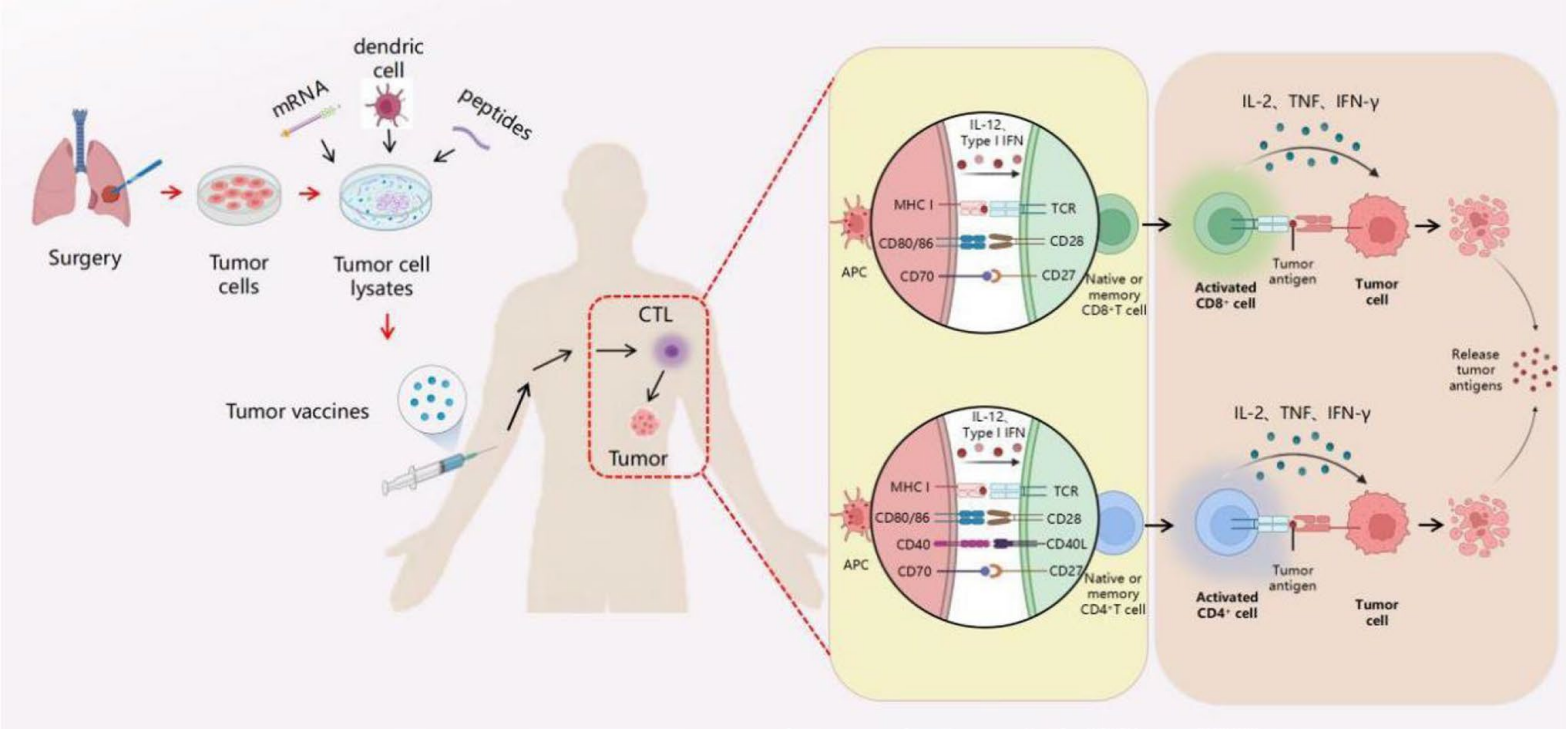
Preparation process of tumor vaccine. The first step in this process involves extracting tumor tissue from a patient with a tumor. This tissue is then divided into tumor lysates. These lysates are loaded into different delivery vehicles and subsequently reinjected into cancer patients. The purpose of this is to stimulate the production of cytotoxic T lymphocytes, which are responsible for killing tumor cells

The concept of 'tumor vaccines' pertains to the inoculation of tumor-associated antigens acquired from tumor tissues or bodily fluids into patients with tumors. This inoculation aims to activate the specific immune response of the body's defense system, eradicating cancerous cells while also managing and addressing cancer [53]. Cancer vaccines can be classified into two main types: preventive vaccines and therapeutic vaccines. Preventive vaccines encompass the Hepatitis B vaccine and the human papillomavirus vaccine [54, 55]. Conversely, therapeutic vaccines can be further categorized into whole-cell vaccines, nucleic acid vaccines, protein/peptide vaccines, and viral vector vaccines [56, 57] (Fig.4). These vaccines are designed to elicit or amplify the immune response against antigens expressed by cancerous cells. This paper primarily aims to conduct a comprehensive review of therapeutic vaccines.

### Tumor vaccines using tumor cells as vectors

Whole-cells vaccines can be classified into two types: whole tumor cells (WTCs) vaccines and dendritic cells (DCs) vaccines. WTCs vaccines involve the selection and processing of autologous or allogeneic tumor cells using different techniques. These cells are genetically modified to create vaccines that can be used for therapeutic purposes in tumor patients. These vaccines can induce specific immune responses as immunogens while maintaining their immunogenicity and non-tumorigenicity [58]. Researchers have conducted studies comparing the effectiveness of WTCs vaccines and split vaccines produced from the same cell lines in inducing specific antibody responses to defined antigens. The results have shown that WTCs vaccines are more successful in eliciting immune responses to cell surface antigens than their soluble or split counterparts [59]. Scientists have explored different approaches to optimize whole-cell vaccines and improve their immunogenicity [60]. These include using intact tumor cells, which are nonviable but maintain their structure, and tumor cell lysates, which are nonviable and have disrupted structures [61]. Specific tumor cell components have also been investigated for their potential to enhance the effectiveness of whole-cell vaccines. A study conducted by Beziaud et al. investigated the combined use of temsirolimus and anticancer vaccines. They discovered that the dual therapy (vaccine + temsirolimus) resulted in the generation of CD8^+^T cells with phenotypic characteristics similar to central memory CD8^+^T cells (CD127^+^CD62L^+^), as opposed to vaccination alone [62]. The clinical trials for tumor whole-cell vaccines have proven to be challenging due to their low immunogenicity; using exogenous adjuvants has been found to enhance the immunity of antigens more effectively. Therefore, a potential alternative has been found in the form of tumor-derived membrane vesicles (TDMVs). TDMVs are noninvasive, chemotactic, and readily accessible, containing diverse tumor-derived substances such as nucleic acids and proteins [63]. Personalized vectors can be created by utilizing the editable properties of cells, which aids in designing personalized vaccines. Recent studies have examined the utilization of TDMVs in personalized immunotherapy and demonstrated the efficient uptake of nano-vaccines encapsulating water-insoluble and water-soluble components of tumor tissue/cells by antigen-presenting cells (APCs) [64]. These nano-vaccines have exhibited improved therapeutic efficacy in melanoma and breast cancer compared to vaccines loaded solely with water-soluble components. Consequently, the primary objective of vaccine development is to enhance the capacity of cellular vaccines to elicit robust and long-lasting immune responses. A pioneering approach in cancer immunotherapy is the utilization of biomaterial-mediated composite cell vaccines. In conclusion, WTCs vaccines have shown promising results in inducing specific immune responses and can be used as therapeutic agents for tumor patients.

DCs, discovered by Ralph Steinman in 1973, are the most powerful APCs recognized [65]. They play a crucial role in initiating and regulating both the innate and adaptive immune responses. The investigation of DCs vaccines carrying tumor antigens is a prominent field in immunotherapy and has tremendous potential for advancement [66]. Currently, multiple strategies have been devised to modify the function of DCs to target cancer progression. These strategies incorporate the usage of immunomodulators, in vitro loading of tumor antigens and neoantigens to mobilize and activate DCs present within the body, and the creation of DC-based cellular vaccines. Up to now, sipuleucel-T is the sole DC-based cancer vaccine authorized by the Food and Drug Administration (FDA) for metastatic castration-resistant prostate cancer (mCRPC) [67]. Nevertheless, its clinical activity is significantly enhanced when combined with other immunomodulatory therapies rather than being utilized as a standalone treatment [68]. According to the United States Clinical Research Database, 615 registered clinical trials are dedicated to exploring the potential of DCs, with 508 explicitly focusing on oncology. In recent years, DC vaccines based on neoantigens have shown clinical success in melanoma and other solid tumors [69]. A single-arm, multicenter pilot study reported the promising efficacy of a personalized neoantigen pulsed dendritic cell vaccine for advanced lung cancer [70]. Using lentiviruses in gene delivery, Leleux et al. found that non-integrated, dendritic cell-targeting lentiviral vectors or vaccines enhance effectors and memory T cell responses [71]. Another phase III clinical trial examined the viability of augmenting the standard of care by adding autologous tumor lysate DC vaccine (DCVax-L) to extend the survival of GBMLGG patients. The findings of this study revealed that the addition of DCVax-L exhibited a favorable safety profile [72]. Although the clinical effectiveness of DC-based therapeutic vaccines has been somewhat restricted thus far, they still offer promise as a possible treatment alternative. Additional research is imperative to determine the immunogenic potential of these cells and ascertain the most effective methods of utilization.

### Peptide vaccines

Peptide vaccines have been widely used to stimulate the immune system and generate specific immune responses in patients. The aim is to enhance the body's ability to fight against tumors and control their growth, spread, and recurrence [73]. Previous studies have focused on improving the efficacy of polypeptide vaccines by exploring different types and forms of adjuvants or antigens. For example, researchers identified immunodominant peptides from a gp100 melanoma-associated antigen and developed a synthetic peptide that increased its binding to specific molecules [74]. This synthetic peptide was then used as a vaccine for patients with metastatic melanoma. In addition, it has been shown that direct intratympanic injection of peptide vaccines can enhance their immunogenicity [75]. Other advancements in peptide vaccines include using long peptides, Toll-like receptor (TLR) agonists, and the combination of systemic therapies, which have shown promise in improving tumor-associated immune function [76]. However, no peptide vaccines are currently available in the market for the treatment of NSCLC. In a recent study, researchers developed a complex with therapeutic functions for preventing and treating epidermal growth factor receptor (EGFR)-positive LUNG. The complex consisted of polyetherimide lipid nanoparticles (PEI-LNP)/siRNA complex (EPV-PEI-LNP-SiRNA) with PD-L1-siRNA and EGFR short peptide/Programmed cell death 1 ligand 1 (PD-L1) double immune-enhancing function [77]. Citrullination is a post-translational modification that occurs in cancer cells during autophagy. This modification induces an immune response by activating peptidyl arginine deiminase (PAD) within APCs and target cells. The immune response is directed toward modified self-epitopes, particularly tumor cells. To enhance this immune response, researchers have introduced standard citrullinated neoantigenic peptides into the immune system. These peptides, such as vimentin and enolase, aim to stimulate a more specific CD4^+^T cell response against NSCLC [78]. In addition, for triple-negative breast cancer, studies have demonstrated the effectiveness of adjuvanted survivin peptide particulate vaccine in the mouse 4T1 tumor line model of triple-negative breast cancer. The results showed that vaccination with survivin peptide antigen was associated with a statistically significant slower primary 4T1 breast tumor growth rate compared with tumors in control mice [79]. However, polypeptide vaccines, which are commonly used in these approaches, have certain limitations. They often exhibit poor metabolic stability and low bioavailability. To address these challenges, researchers have explored the use of modified peptides. These modified peptides can potentially improve the vaccines' stability and bioavailability. Different strategies have been employed, including combining the modified peptides with immune adjuvants, combining them with DCs, creating multi-antigen branch peptides, or developing multi-epitope superposed peptides. Despite the advancements made, there are still drawbacks associated with polypeptide vaccines. However, the ongoing advances in technology hold promise in overcoming these limitations. With further development, polypeptide vaccines could offer an alternative approach to the biological treatment of tumors.

### Nucleic acid vaccine

Deoxyribonucleic acid (DNA) vaccines have been proven to activate the immune system and induce immune responses effectively. They have many advantages, such as specificity, safety, stability, low cost, and the ability for post-translational modification [80]. The first clinical trials of a plasmid DNA vaccine were conducted twenty years ago, evaluating its safety, feasibility, and biological efficacy [81]. Different strategies have been employed to enhance the effectiveness and immunogenicity of DNA vaccines in clinical trials, confirming this platform's safety and tolerability. Various aspects of DNA vaccines, from basic research to clinical evaluation, have been thoroughly examined. A longitudinal immunologic analysis has been conducted to guide future clinical trials in developing optimal vaccination regimens [82]. This analysis's primary objective was to answer various crucial inquiries: (1) Does the immune response transpire early during the immunization process, or is it solely triggered after receiving multiple immunizations, thereby indicating the necessity for ongoing immunization? (2) Is there a delayed immune response occurring several months following the initial course of immunization? (3) Once stimulated, does the immune response exhibit durability? (4) Is the emergence of an immune response correlated with a potential clinical reaction? To explore these queries, a computer-based simulation methodology was implemented to identify distinctive cytotoxic T lymphocyte (CTL) epitopes and create a multi-epitope DNA vaccine capable of effectively instigating desirable immune responses against cancers with overexpression of Wilms tumor gene 1 (WT1) [83]. This innovative approach has revealed promising outcomes, validated by Khalili et al., who effectively employed computer simulation technology for vaccine design and elicited favorable anti-tumor immune responses [84]. Nevertheless, the application of DNA vaccines in cancer treatment remains confined. In recent years, researchers have been focusing on maximizing the effectiveness of DNA vaccines. One approach is to combine them with other therapies or adjust adjuvants. For example, Pan et al. developed a cytotoxic T lymphocyte-associated antigen-4 (CTLA-4)-PD-L1 DNA cancer vaccine and tested it in a rat model of HCC induced by thioacetamide [85]. The vaccine successfully reduced tumor growth, demonstrating its therapeutic efficacy. Another study explored a DNA DCs vaccine, a molecular nanostructure composed of adjuvant DNA strands that split into multiple DNA branches and contain different amounts of bound peptide antigens. This vaccine showed promising results regarding how DCs take it up, its ability to activate the immune system, and its potent anticancer effects [86]. Future research should focus on several key areas to ensure the success of anti-tumor DNA vaccines. One area of emphasis should be targeting the inflammatory signaling cascade using small molecules. This would help enhance the immune response and improve the efficacy of the DNA vaccine. Additionally, researchers should aim to select more immunogenic tumor antigens, as this can further improve the vaccine's ability to target and kill cancer cells. Another vital aspect to consider is the development of improved delivery systems for the DNA vaccine. This would ensure the vaccine effectively reaches the targeted cells and maximizes its therapeutic potential. Lastly, combination therapy should be explored further. Researchers may enhance the overall anti-tumor response and improve patient outcomes by combining DNA vaccines with other treatments, such as immunotherapies or traditional chemotherapy.

The TAA-focused messenger RNA (mRNA) vaccines, developed by both Moderna and BioNTech, have displayed remarkable efficacy, ease of development, and absence of viral components harmful to cells and pathogens [87]. Trials conducted on patients with solid tumors have unequivocally demonstrated the robust anti-tumor immune response generated by these mRNA vaccines [88]. Previous investigations have sought to boost the immunogenicity of nucleic acid vaccines by facilitating their capacity for self-replication [89]. To enhance human epidermal growth factor receptor 2 (HER-2) DNA cancer vaccines, Wang et al. investigated the possibility of employing RNA interference (RNAi) to suppress FOXO3 in antigen-presenting cells as an adjunct treatment [90]. The pathway known as 4-1BB/4-1BB ligand (4-1BBL) plays a critical role in the immune response, tumor immunity, and autoimmune diseases by transmitting T-cell costimulatory signals. Consequently, scientists have devised an experimental design that involves transfecting a DC vaccine with total RNA extracted from GC cells that carry the 4-1BBL gene in a controlled laboratory setting. This study aimed to evaluate the potential anti-tumor effect of this modified DC vaccine on the GC of mice [91]. In recent decades, various delivery systems have been created to safeguard mRNA molecules from degradation caused by external or internal ribonucleases (RNases). The transport of RNA vaccines using non-viral vectors has demonstrated substantial promise in treating tumors. Vormehr et al. successfully developed a therapeutic cancer vaccine using a liposome formulation containing RNA that encodes tumor antigens (RNA-LPX) [92]. This delivery system has exhibited the capacity to elicit robust T cells responses in both mice and humans. Among the different non-viral vectors, lipid nanoparticles (LNPs) have shown the most potential for delivering mRNA. Another investigation has effectively produced a novel circular RNA (circRNA) vaccine platform by enclosing circles carrying antigen-encoded information within LNPs [93]. This approach resulted in a vigorous activation of innate and adaptive immunity, leading to notable anti-tumor effects in mouse tumor models. Notably, the FDA has recently acknowledged the breakthrough therapy designation for a cancer vaccine named mRNA-415/V940, intended for use as an adjuvant therapy in patients with completely resected, high-risk melanomas [94]. In March of that same year, the NMPA sanctioned the 'LK101 injection' as a cancer tumor vaccine. This groundbreaking vaccine, known as the first personalized tumor neoantigen vaccine authorized by the NMPA in China, is also recognized as the inaugural fully personalized mRNA editing product to receive approval for clinical trials. To summarize, mRNA represents a potent and adaptable foundation for tumor vaccines, and the achievement of its successful clinical application will significantly augment our capacity to combat cancer. Future research endeavors should prioritize comprehending and utilizing the paradoxical aspects of mRNA's innate immunity. Additionally, efforts should be directed toward enhancing the efficacy of antigen expression and presentation through state-of-the-art and well-tolerated delivery systems and modifying mRNA structures to extend and regulate the duration of the term.

## Monoclonal antibody therapy

**Fig. 5:**
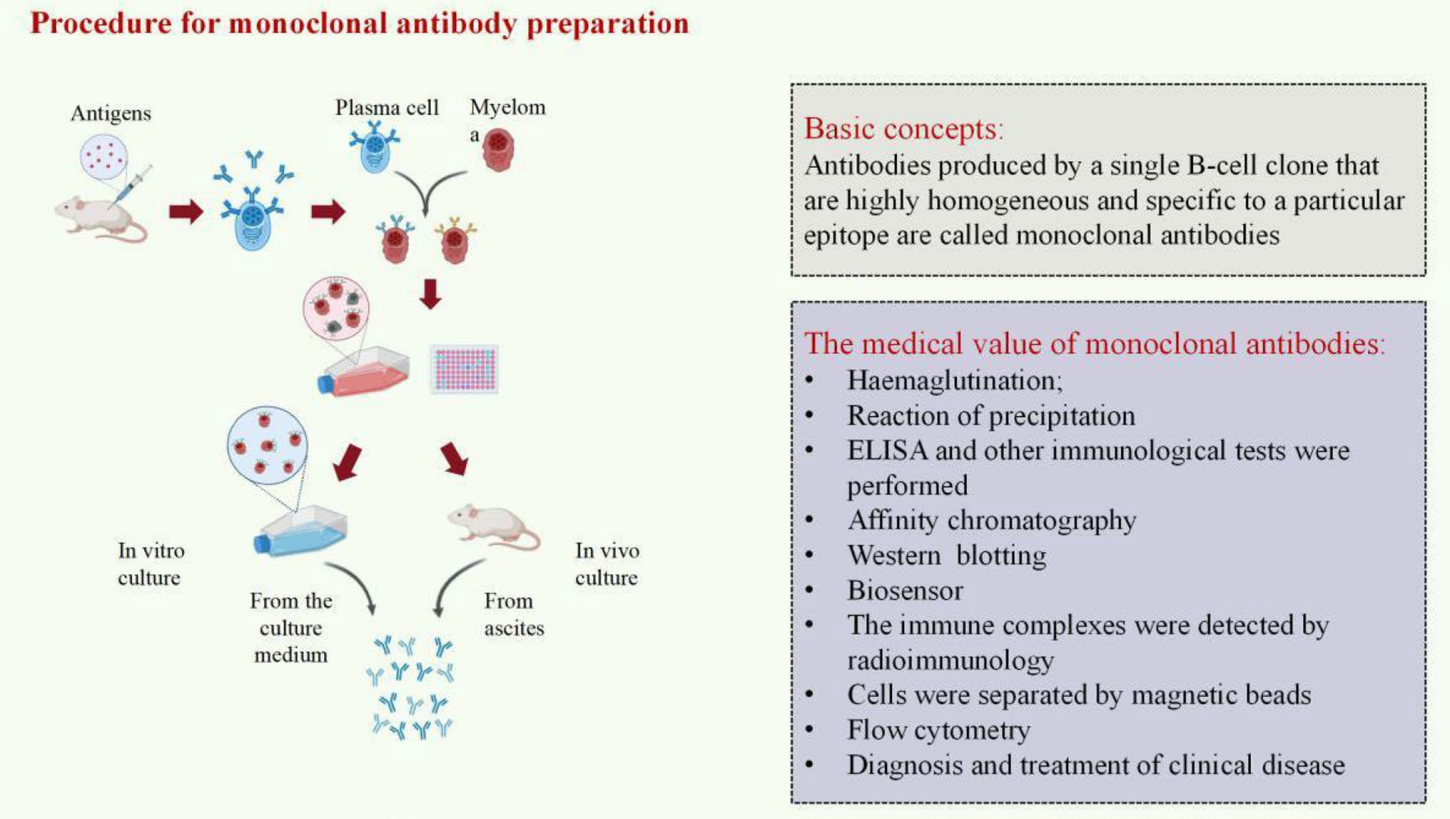
Process of monoclonal antibody preparation. Initially, mice are immunized with the antigen, which then reaches peripheral immune organs via blood or lymph circulation. This process stimulates the cloning, activation, proliferation, and differentiation of corresponding B lymphocytes into sensitized B lymphocytes. Subsequently, the mice are sacrificed, and the spleens are aseptically removed. Spleen cells are prepared, and a spleen cell suspension is created. These lymphocytes can then fuse with myeloma cells, resulting in the formation of hybridoma cells. Finally, the hybridoma cells are selected for further study

In 1975, Kohler et al. made a breakthrough in mAb research by using hybridoma technology to obtain the first mouse-derived mAb. This was a significant milestone in the field [95] (Fig.5). Monoclonal antibodies include a Fab tail that binds to tumor antigens and an Fc tail that binds to immune cell surface receptors. In the presence of monoclonal antibodies, two cells bind through complement-mediated cytotoxicity in an antibody-dependent manner, and the cytotoxic effects of these cells kill tumor cells [96, 97]. With the advancement of immunological theory and biotechnology methods in recent years, targeted therapy using mAbs and biological therapy utilizing immune cells have become widely adopted in tumor treatment [98]. Over three decades ago, researchers found that mouse–human chimeric mAbs targeting carcinoembryonic antigen (CEA) could accurately locate tumor sites [99]. Moreover, applying macrocyclic radioactive metal complexes in conjunction with mAbs has exhibited promise in diagnosing and treating human cancers [100]. The strategies employed for targeted therapy against malignant tumors by utilizing mAbs consist of (1) directing attention toward surface antigens found on malignant tumor cells or tumor-promoting cytokines and their corresponding receptors to achieve an anti-tumor impact; (2) facilitating immune cell therapy by activating or inhibiting tumor-specific T lymphocytes. Notably, mAbs have demonstrated remarkable efficacy in clinical settings, establishing themselves as a potent weapon against diverse cancers. Over the past few years, scientists have significantly enhanced the effectiveness of mAbs in treating cancer. SNAP-tag, a modified form of the human enzyme O6-alkylguanine-DNA alkyl transferase, has been genetically fused with antibody fragments to create fusion proteins. These fusion proteins can be easily labeled with benzyl guanine-modified payloads for targeted delivery. In a study by Malindi et al., the researchers examined the advantages and limitations of these techniques and their combined impact on improving photodynamic therapy for melanoma [101]. They presented a case study featuring a postmenopausal Chinese woman with metastatic disease. The patient responded positively to advanced treatment using margetuximab in combination with chemotherapy. This patient had been diagnosed with estrogen receptor (ER), progesterone receptor (PR), and HER-2+ invasive ductal carcinoma (ICD) [102]. Another clinical trial conducted on sixty-two Italian patients with refractory rat sarcoma (RAS) wild-type metastatic colorectal cancer (MCRC) evaluated the efficacy of panitumumab when combined with trifluoro uridine+pyrazine anti-EGFR as rechallenge therapy. The results indicated that combining Panitumumab and trifluorouridine+tipiracil improved progression-free survival (PFS) at 6 and 12 months compared to trifluorouridine+tipiracil alone [103]. Currently, several monoclonal antibodies, including rituximab, trastuzumab, pembrolizumab, ocrelizumab, bevacizumab, tislelizumab, sintilizumab, and nivolumab, are employed as anti-tumor medications in the realm of clinical practice (Table 1) At present, scientists are still developing new products in order to bring more hope to patients. Monoclonal antibodies have become a magical weapon to change the rules of cancer treatment, and it is also one of the most effective weapons to help us eliminate cancer.

**Table 1:** Monoclonal antibodies drugs are common in malignant tumors

Name	Target	Type	Indication
Rituximab	CD20	Chimerism	Hematological malignancies
Brentuximab	CD30	Chimerism	Hodgkin lymphoma
Trastuzumab	HER-2	Personalization	Breast cancer
Obinutuzumab	CD20	Personalization	Chromic lymphocytic leukemia
Bevacizumab	VEGF	Personalization	Colon cancer, lung adenocarcinoma, etc.
Pertuzumab	HER-2	Personalization	Breast cancer
Nivolumab	PD-1	Personalization	Melanoma, etc.
Pembrolizumab	PD-1	Personalization	Melanoma, etc.
Cetuximab	EGFR	Chimerism	Colon cancer

## *Cancer* stem cell therapy

Cancer stem cells (CSCs) possess the unique ability to self-renew and have a high potential. Like normal stem cells, they can protect themselves, allowing them to resist conventional tumor treatments and leading to tumor recurrence and metastasis [104]. In a groundbreaking study in 1997, Bonnet et al. found that a subset of tumor cells with specific surface markers, CD34^+^ and CD38^-^, could be transplanted into immunodeficient mice to produce metastatic leukemia [105]. This discovery confirmed the existence of CSCs in human tumors for the first time. For more than 20 years, researchers have been working to harness CSCs for cancer treatment. As a result, targeting CSCs has become a promising approach to cancer treatment. Angiogenesis, the formation, and maintenance of blood vessels, plays a vital role in tumor growth. Angiogenesis, which refers to the formation and maintenance of blood vessels, is known to play a critical role in the growth of tumors. According to the U.S. Clinical Trials Database, there are currently multiple clinical trials of CSCs for treating hematological and solid tumors (Table 2). Bao et al. have found that the distinct characteristics of the CSCs subgroup contribute to the development of malignant tumors and could be targeted in anti-angiogenesis therapy [106]. Recent studies have shown that CD13 can be a marker for semi-quiescent CSCs in human HCC cell lines and clinical samples [107]. This information provides valuable insights into identifying and targeting CSCs for more effective treatment strategies. Targeting CSCs has potential as a therapeutic approach for treating cancer. There is increasing evidence that CSCs interact with immune cells in the tumor microenvironment. These interactions involve tumor-associated macrophages (TAMs), myeloid-derived suppressor cells (MDSCs), and T cells. The immune cells play a crucial role in maintaining the stemness of CSCs and survival. Researchers have found a negative correlation between cancer stemness and anticancer immunity. For example, Krishna et al. demonstrated the association between cancer and complete subsidence using high-dimensional analysis of human ACT products. They observed that tumor-infiltrating lymphocytes (TILs) had a persistent memory progenitor cell phenotype in CD39^-^ dry samples, which was linked to poor persistence of TILs and the terminal differentiation of CD39^+^ state [108]. Human stem cells are attracted to tumors through chemoattractants secreted by cancer cells, including various cytokines (such as Stem cell factor and monocyte chemoattractant protein-1) and growth factors (such as Hepatocyte growth factor and vascular endothelial growth factor) [109]. In a study to find effective anti-tumor therapies for GC, researchers used neural stem cells expressing cytosine deaminase and interferon beta (HB1. F3. CD. IFN -β). These cells were able to convert non-toxic 5-FC into 5-FC, a compound with cell toxicity that can exert an anti-tumor effect. Additionally, the cells secreted IFN-β, further contributing to the anti-tumor effect [110]. One of the challenges in treating cancer is the ability of CSCs to inhibit the immune response. CSCs recruit immunosuppressive cells, such as TAMs and regulatory cells (Tregs), which promote the establishment of an immunosuppressive TME. Moreover, CSCs impair the function of natural killer (NK) cells by expressing specific ligands. Understanding the molecular mechanism underlying the anti-tumor immune response is crucial as it can help develop new and more effective anti-tumor therapies.

**Table 2:** The role of cytokines in the tumor microenvironment

Antitumor effect	
Direct tumor suppression	TGF- β, lFN**-** γ
Enhanced the cytotoxic activity of lymphocytes	IFN**-** γ**, **IL-2, 1L-12, IL-15
Enhanced the cytotoxic activity of myeloid cells	IFN**-** γ

Tumor promoting effect	
Promote tumor cell survival, stem cell and proliferation	TGF- β, TNF, IL-l β, CXCL-12
Immunosuppression	IL-10, IL-4, TGF**-** β
Dysregulation of cytokine networks and promoting of tumor spread	TNF, IL-1α, IL-1β, IL-6
Promote EMT, migration and invasion of tumor cells	TGF**-**β, various cytokines
Promote abnormal ECM production and	
Immunosuppression by tumor-associated	TGF-β, IL-1β
Fibroblasts	
Promote angiogenesis	TNF, IL-6, various cytokines

## Cytokines therapy

Cytokines are essential molecules that regulate the immune system and influence various cell types and activities [111]. They include significant cytokines like interleukins (ILs), interferons (IFNs), tumor necrosis factors (TNFs), chemokines (CKs), and growth factors (GFs), which are crucial for maintaining cellular function in both normal conditions and diseases. Furthermore, cytokines play a vital role in communication between cells within the TME. In relation to their impact on neoplasms, the growth inhibition of tumor cells has been directly observed with interferon and TGF-β. Moreover, there has been evidence suggesting that IFN-γ, IL-2, IL-12 and IL-15 augment the cytotoxic function of lymphocytes or bone marrow cells while restraining cell proliferation. Conversely, TGF-β, TNF, IL-1β, and chemokine ligand 12 (CXCL12) are credited with fostering the survival and propagation of malignant cells. TNF and IL-6, in particular, are capable of disrupting cytokine regulation and instigating inflammation within tumors. Furthermore, immunosuppression has been attributed to IL-10, IL-4, and TGF-β. TNF, IL-6, and a variety of chemokines also contribute to the promotion of angiogenesis (Table 2). In recent years, significant research has focused on cytokines and their receptors as potential targets or therapies for tumors. Promising preclinical evidence suggests that cytokines like IFNs and ILs can enhance growth inhibition and promote immune responses [112]. Efforts have also been made to develop therapeutic strategies to counteract specific cytokines' inflammatory and tumor-promoting effects, such as TNF, IL-1β, and IL-6. One example of a cytokine therapy approved by the FDA is high-dose intravenous recombinant IL-2, which has been used to treat interstitial renal cell carcinoma (RCC) and melanoma[113]. However, this treatment has limitations due to its short half-life and potential negative effects on T cells, including Tregs. These complications restrict its use in clinical settings. In recent years, there has been significant advancement in enhancing the longevity of IL-2 and reducing complications through various cutting-edge technologies. A recent study examined the efficacy of IL-2 biological preparation Bempegaldesleukin, which contains polyethylene glycol (PEG), in treating patients with metastatic melanoma. The findings demonstrated the effectiveness of Bempegaldesleukin in activating CD8^+^ T cells and NK cells. Additionally, it was observed that Bempegaldesleukin could prolong the half-life of IL-2 to approximately 20 hours [114]. Combined with ICIs like nivolumab, this treatment induces tumor immune responses in various cancer types, including melanoma, RCC, NSCLC, urothelial cancer (UCC), and triple-negative breast cancer (TNBC). Objective response rate (ORR) ranges from 33% to 75%, with a low occurrence (around 20%) of treatment-related severe adverse events [115–117]. Because of its short half-life and high incidence of therapeutic adverse events, IFN-α monotherapy has been rarely used in solid tumors, but it does have considerable clinical activity and utility in hematological tumors. In the field of diseases characterized by abnormal cell growth, certain conditions such as bone marrow fibrosis (BMF), primary thrombocytosis (PT), chronic myeloid leukemia (CML), follicular lymphoma (FL), and inhibitor host disease resistance have been identified [118, 119]. Bhat et al. shed light on the understanding and functional implications of cytokines and chemokines dysregulation in aggressive endometrial cancer (EC), emphasizing the complex nature of cytokines–chemokines networks and their impact on treatment outcomes [120]. Recent research has made strides in developing a novel immune cytokine, IAP0971, that targets explicitly programmed death 1 (PD-1) and integrates the IL-15/IL-15Rα complex for cancer immunotherapy [121]. However, efforts to modulate individual cell factors to either suppress or boost tumor growth have shown limited efficacy in achieving long-lasting activity against tumors, primarily due to the constraints associated with cytokine therapy. These limitations encompass a short half-life, a narrow therapeutic window, and an augmentation of immune-enhancing mechanisms. Cancer immunotherapy has demonstrated the ability of a variety of nanomaterials to administer cytokines or regulate their expression in tumor cells directly. Notable nanomaterials in this regard encompass polylactic acid-glycolic acid copolymer (PLGA), nanomaterials derived from chitosan, silica nanoparticles, calcium carbonate/calcium phosphate nanoparticles, and other groundbreaking nano-delivery systems [122–125]. In forthcoming research, the emphasis should be on merging cytokine-based therapy with additional immunotherapies and/or modifying cytokines to alter their pharmacokinetics and binding affinity. This strategy holds promise for minimizing toxicity, evading the introduction of immunosuppressive factors, and enhancing treatment outcomes.

## Adoptive cell transfer therapy

**Fig. 6:**
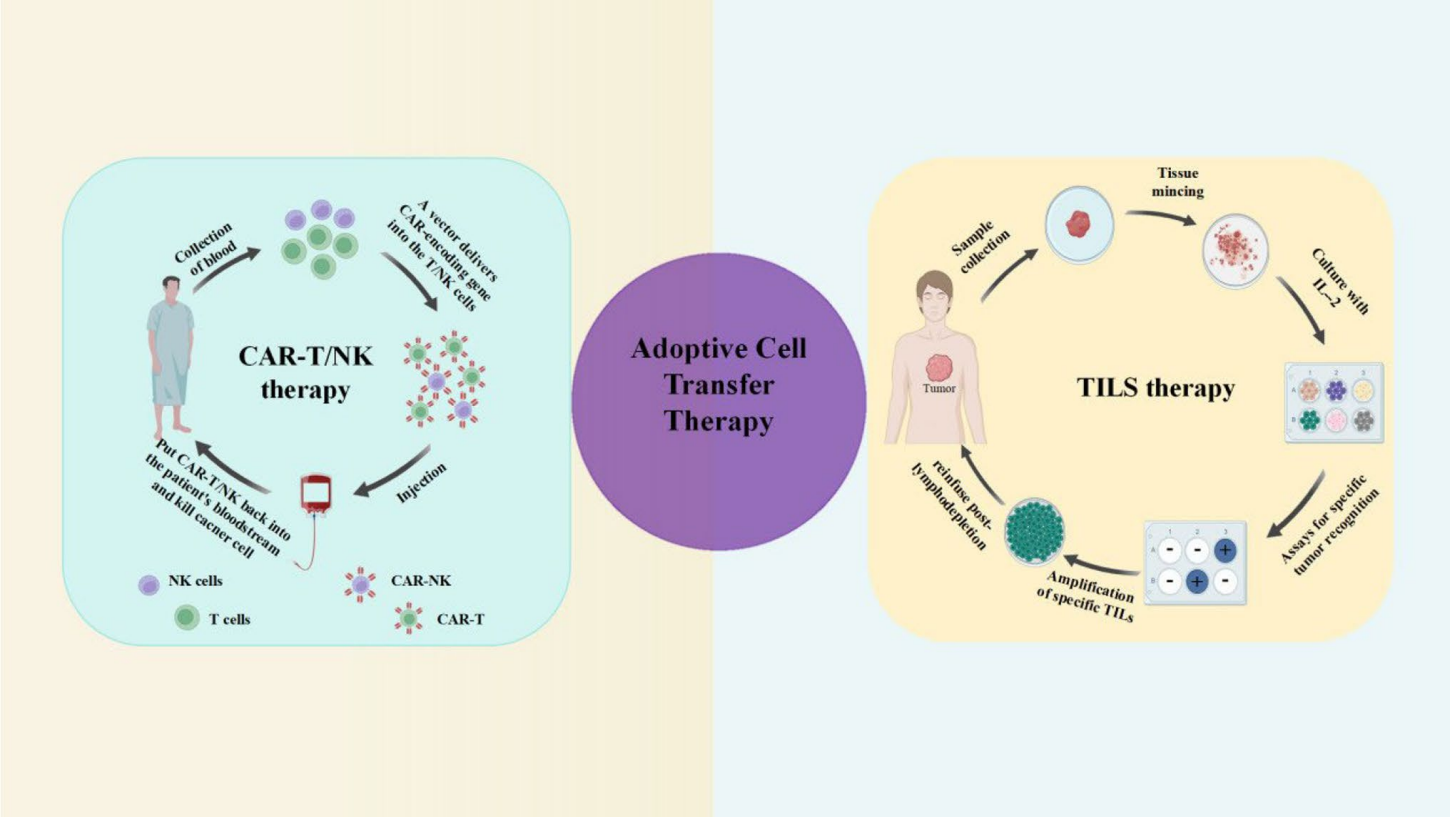
Types of adoptive cell transfer therapies. ACT is a treatment strategy that involves gathering immune cells from the patient, specifically killer T cells. These cells are then nurtured and altered outside the body to enhance their capacity to specifically eliminate cancerous cells. Following this manipulation, the modified cells are reintroduced into the patient's system to effectively eradicate the tumor cells

Adoptive cell transfer therapy (ACT),is a treatment strategy that involves gathering immune cells from the patient, specifically killer T cells. These cells are then nurtured and altered outside the body to enhance their capacity to specifically eliminate cancerous cells. Following this manipulation, the modified cells are reintroduced into the patient's system to effectively eradicate the tumor cells. The field of adoptive immune cell therapy encompasses a range of techniques, including engineered T cell receptor (TCR) therapy, chimeric antigen receptor (CAR) therapy, natural killer (NK) cell therapy, and tumor-infiltrating lymphocyte (TIL) therapy (Fig.6).

### Chimeric antigen receptor T-cell immunotherapy

Chimeric Antigen Receptor T-cell (CAR-T) immunotherapy utilizes gene engineering technology to activate and multiply the T cells in the body in vitro. These genetically modified T cells are then reintroduced into the patient's body to target and eradicate cancer cells specifically [126]. CAR is a membrane protein that functions as a recombinant receptor. It is composed of interconnected protein functional domains, which contribute to its flexibility and specificity in antigen recognition. These functional domains consist of a single-chain antibody domain (scFv) responsible for recognizing and binding TAA, a hinge region, a transmembrane domain, a costimulatory domain (CD28 or 4-1BB), and an intracellular T cell activation domain[127, 128]. The first generation of CARs exhibited limited efficacy in clinical trials due to slow expansion and short durability. However, continuous optimization and improvements have led to the remarkable clinical success of CARs in diagnosing and treating various tumors, particularly malignant blood system tumors. In 2017, the FDA approved Tisagenlecleucel and Axicabtagene ciloleucel (Axi-CEL), the first CD19 targeted second-generation CAR-T cells products, for treating refractory/relapsed B-cell lymphoma (R/R DLBCL) in individuals below 25 years old [129]. Kandalaft et al. presented a phase study proposal to evaluate the viability, safety, and initial effectiveness of FRα-redirected CAR-T cells incorporating the CD137 (4-1BB) costimulatory domain [130]. These CAR-T cells would be administered after lymphodepletion to address recurrent ovarian cancer (ROC). Recent research has demonstrated the effectiveness of CAR-T immunotherapy in treating solid tumors. Sridhar et al. suggest that delivering CAR-T cells regionally could be a promising strategy for solid tumor immunotherapy [131]. They believe further exploration of this technique through clinical trials in HNSC, mesothelioma (MESO), and peritoneal carcinomatosis (PC) is warranted. Additionally, the combination of Clustered Regularly Interspaced Short Palindromic Repeats (CRISPR/Cas9) technology and CAR-T cell immunotherapy has shown potential for improving the efficiency and safety of CAR-T cells. Researchers have discussed the mechanism and treatment of CRISPR/Cas9 technology, its accuracy, and the application of CRISPR technology in producing genetically modified CAR-T cells to enhance their anti-tumor effect [132]. Numerous clinical studies have identified specific targets, such as mesothelin (MSLN), CD133, Prostate stem cell antigen (PSCA), Claudin 18.2, and HER-2, for CAR-T immunotherapy in pancreatic cancer (PCA). Among these, Claudin 18.2 and MSLN have gained significant popularity [133, 134]. However, treating solid tumors like COAD with CAR-T cells faces several challenges. These challenges include limited transport within tumor tissues, an immunosuppressive TME, tumor heterogeneity, and adverse reactions during and after CAR-T cell immunotherapy. Li et al. propose using novel tumor-specific antigens as targets for more effective and safer CAR-T cells immunotherapies and antibody-drug conjugates (ADCs) to overcome these limitations [135]. Although promising in tumor treatment, CAR-T therapy has significant adverse reactions, including CAR-related hematologic toxicity, targeted nontumor toxicity, cytokine release syndrome (CRS), and immune effector cell-associated neurotoxicity syndrome (ICANS). While tocilizumab has shown effectiveness in preventing CRS, it fails to address the risk of fatal neurotoxicity such as meningitis [136–138]. In mice, Anakinra, an IL-1 receptor antagonist, has demonstrated the ability to reduce CRS and fatal neurotoxicity by disrupting the IL-1 pathway. Clinical trials are underway to test Anakinra's potential in preventing neurotoxicity in patients receiving CAR T cell treatment [139]. The challenge lies in developing new strategies to prevent or alleviate CRS and neurotoxicity, hindering the widespread clinical use of CAR-T. Ongoing studies, both domestically and internationally, aim to identify effective specific antigens, enhance T-cell production, and decrease the associated side effects and costs of treatment.

### Chimeric antigen receptor nk-cell immunotherapy

NK cells are derived from lymphoid stem cells discovered in the bone marrow of humans. Unlike T and B cells, NK cells belong to a specific subset of lymphocytes proficient in effectively eliminating tumor cells and virus-infected cells without needing prior sensitization [140]. When NK cells are activated, they can produce and release various cytokines, regulate immune and hematopoietic functions, and directly eradicate target cells. The development of CAR technology has introduced the possibility of utilizing CAR-NK cells as a prospective treatment option. The distinct innate characteristics of NK cells enable CAR-NK cells to avoid inducing a potent cytokine storm during treatment, unlike CAR-T cells, which potentially renders them safer for clinical applications [141]. Nevertheless, it is crucial to acknowledge that CAR-NK cells exhibit a shorter lifespan within the body when compared to CAR-T cells. Although this may result in reduced efficacy, it also mitigates potential risks associated with the long-term presence of genetically modified immune cells in the body [142]. Xiao et al. devised a CAR by combining the extracellular domain of the natural killer group 2-member (DNKG2D) receptor, inherent to NK cells, with DAP12 [143]. The purpose of this fusion was to enhance NK cells' response to tumors. Hu validated utilizing tissue factor targeted as a CAR-NK immunotherapy to treat TNBC efficiently [144]. However, additional preclinical investigations and prospective studies involving TNBC patients are necessary to explore novel targets. In a 2017 phase I trial, eight patients with various types of cancer, including PCA, who tested positive for PD-1 and mucin 1 (MUC1) were treated with CAR-NK cells that targeted both MUC1 and PD-1. Throughout the treatment and follow-up period, all seven patients remained stable and did not experience any serious adverse events. Subsequent phase II clinical trials have been conducted to gather additional clinical research data [145]. CAR-NK immunotherapy shows great promise in cancer treatment, with roundabout guidance receptor 1 (ROBO-1) being another target of interest. Currently, three ongoing phase I/II clinical trials evaluate CAR-NK cell therapy's safety and effectiveness directed at ROBO-1 in PCA and other solid tumors [146]. These results are highly anticipated as immunotherapy has emerged as a promising new approach to cancer treatment. With the exceptional anti-tumor characteristics of NK cells, CAR-NK immunotherapy has the potential to be a significant breakthrough in the treatment of malignant tumors.

### Tumor-infiltrating lymphocytes immunotherapy

TILs immunotherapy is a process that involves isolating TILs from tumor tissue, culturing them in vitro, expanding their numbers, and reintroducing them into the patient's body. This approach has shown promise in treating metastatic melanoma, as summarized in previous studies [147]. Radvanyi has addressed several important questions regarding TILs, including their mechanism of action, the subsets of active T cells, the necessity of lymphoablative preconditioning, predictive biomarkers, the potential of combination therapy such as checkpoint blockade, the recognition of mutated antigens by TILs, and the development of TILs for nonmelanoma indications [148]. In addition to melanoma, TILs have also been evaluated in BC. Denkert et al. conducted a comprehensive evaluation of TILs in 3771 cases of BC from six clinical trials [149]. Their study assessed the relevance of TILs for pathological complete response (pCR), disease-free survival (DFS), and overall survival (OS) in different molecular subtypes of BC. Overall, these studies highlight the potential benefits and complexities of TILs therapy. Understanding the mechanism of action, identifying predictive biomarkers, and exploring the role of combination therapy are crucial for developing effective TILs-based treatments for various types of cancer. Furthermore, evaluating TILs in different molecular subtypes of BC provides valuable insights into their relevance as potential indicators of treatment response and prognosis in this specific context. LN-145, developed by Iovance, is an autologous TILs-based therapy that has shown positive results in patients with advanced cervical cancer (CCA) (NCT03108495) The data show an ORR of 44%, indicating that nearly half of the patients experienced a positive response to the treatment. Moreover, no significant side effects were observed, indicating that LN-145 is well-tolerated by patients. These findings have led to LN-145 being granted breakthrough treatment designation by the FDA, making it the first cellular immunotherapy for a solid tumor to receive this distinction. This recognition underscores the potential of LN-145 as an effective treatment option for advanced CCA. Furthermore, LN-145 has also shown promise in the treatment of NSCLC [150]. In the trial subjects, the ORR was 21.4%, indicating that a significant proportion of patients experienced a positive response to the therapy. This demonstrates that LN-145 may be a viable treatment option for NSCLC patients who have not responded to conventional therapies. In addition to LN-145, Iovance has also developed LN-144, another autologous TILs-based therapy specifically for melanoma. In a trial involving patients with advanced melanoma and PD-1, LN-144 demonstrated an impressive disease DCR of 80% [151]. This means that the therapy was able to effectively control the progression of the disease in the majority of (in most) patients. The treatment has proven to be successful in managing the progression of the disease in a significant portion of patients. Furthermore, even patients who did not respond to ICIs, particularly those who were PD-L1 negative, still experienced benefits from TILs immunotherapies. This highlights the potential of TILs as a treatment option for melanoma patients who have not responded to other therapies. Overall, the results of these trials suggest that T cells-based adoptive cellular therapies, such as LN-145 and LN-144, can potentially be effective treatments for solid tumors. These therapies have shown promising ORR and disease control rates, indicating their ability to impact patient outcomes positively. Furthermore, the fact that LN-145 has received breakthrough treatment designation from the FDA signifies its potential as an innovative and vital treatment option. Further research and clinical trials will be necessary to understand and optimize these therapies' potential fully, but their initial success is undoubtedly encouraging. Through continuous improvement and development, TIL therapy will eventually become a new weapon for humans to fight cancer.

## Summary and outlook

In recent years, the treatment of malignant tumors has witnessed a growing prominence of tumor biotherapy. The increasing relevance of tumor immunotherapy can be attributed to the emergence of fresh technologies, techniques, and the integration of varying disciplines. Nevertheless, managing adverse events and ensuring consistent therapeutic efficacy present significant challenges, considering tumor heterogeneity and individual immune disparities. Several obstacles need to be surmounted to optimize the effectiveness of tumor immunotherapy. These include the identification of the suitable target molecule for each tumor, minimizing therapy's toxic side effects, and strategically handling associated costs. Encouragingly, genetic engineering has made remarkable strides in addressing these hurdles, demonstrating immense potential in eradicating tumors and offering novel treatment alternatives for patients with progressive malignancies. Such a comprehensive strategy holds promise in partially transcending the restrictions of existing tumor immunotherapy approaches. In the future, the progress and integration of tumor immunology, computer science, bioinformatics, and correlated disciplines will further propel the advancement of tumor immunotherapy. By harnessing novel theories and technologies, innovative surgical methodologies and treatment modalities can be formulated to heighten the immune system's capacity to obliterate tumor cells safely and effectively, ultimately accomplishing the objective of long-lasting cures with minimal toxicity. Therefore, we can anticipate noteworthy strides in treatment techniques and modalities, ultimately conferring benefits upon cancer patients through an expanded range of options and improved outcomes.

## Data Availability

No data were used for the research described in the article.
